# Loads of unconscious processing: The role of perceptual load in processing unattended stimuli during inattentional blindness

**DOI:** 10.3758/s13414-020-01982-8

**Published:** 2020-02-04

**Authors:** Giulia Pugnaghi, Daniel Memmert, Carina Kreitz

**Affiliations:** Institute of Exercise Training and Sport Informatics, German Sport University Cologne, Am Sportpark Müngersdorf 6, 50933 Köln, Germany

**Keywords:** Inattentional blindness, Unconscious processing, Attention, Perceptual load model, Interference

## Abstract

Inattentional blindness describes the failure to detect an unexpected but clearly visible object when our attention is engaged elsewhere. While the factors that determine the occurrence of inattentional blindness are already well understood, there is still a lot to learn about whether and how we process unexpected objects that go unnoticed. Only recently it was shown that although not consciously aware, characteristics of these stimuli can interfere with a primary task: Classification of to-be-attended stimuli was slower when the content of the task-irrelevant, undetected stimulus contradicted that of the attended, to-be-judged stimuli. According to Lavie’s perceptual load model, irrelevant stimuli are likely to reach awareness under conditions of low perceptual load, while they remain undetected under high load, as attentional resources are restricted to the content of focused attention. In the present study, we investigated the applicability of Lavie’s predictions for the processing of stimuli that remain unconscious due to inattentional blindness. In two experiments, we replicated that unconsciously processed stimuli can interfere with intended responses. Also, our manipulation of perceptual load did have an effect on primary task performance. However, against our hypothesis, these effects did not interact with each other. Thus, our results suggest that high perceptual load cannot prevent task-irrelevant stimuli that remain undetected from being processed to an extent that enables them to affect performance in a primary task.

Surprisingly often we are unaware of clearly visible stimuli in our direct view simply because they appear unexpectedly and outside our attentional focus, a phenomenon called inattentional blindness (Mack & Rock, 1998). Inattentional blindness paradigms are thought to be especially rigorous measures of attentional capture as critical stimuli appear completely unexpectedly, and thus, voluntary attention cannot be directed towards its detection (e.g., New & German, 2015). Therefore, all factors influencing the processing of the critical stimulus and its crossing of the threshold of awareness rely on involuntary distribution of attention. By now, a lot of research has been conducted to unravel the factors that determine whether or not inattentional blindness occurs — namely, whether or not an unexpected object crosses the threshold of awareness (i.e., was reported by the participants; Kreitz, Furley, Memmert, & Simons, 2016; Mack, Pappas, Silverman, & Gay, 2002; Most et al., 2001; Simons & Jensen, 2009).

This binary view on awareness is a general pattern in inattentional blindness research, while much less is known about the fate of those stimuli that remain undetected due to inattentional blindness. One theoretical framework that distinguishes different types of processing and provides distinguishable predictions for those types is the global neuronal workspace hypothesis (Dehaene, Changeux, Naccache, Sackur, & Sergent, 2006). Among other things, this framework predicts substantial processing of stimuli that have sufficient bottom-up strength but remain undetected due to a lack of attentional amplification (preconscious processing^1^), as is the case in inattentional blindness paradigms. And, indeed, the existing body of research on that topic indicates that there can be perceptual processing of unexpected objects even if they are not consciously noticed and therefore cannot be reported. In their seminal book, Mack and Rock (1998) report experiments in which they investigated whether features of an undetected object could prime following responses. They found evidence for perceptual processing in the absence of awareness; in a stem completion task that immediately followed an inattentional blindness task, participants were significantly more likely to complete the stem in accordance with the word that was unexpectedly presented in the inattentional blindness task. Other research has shown that behavioural responses can be influenced by grouping processes that occur completely outside of our awareness (Lamy, Segal, & Ruderman, 2006; Moore & Egeth, 1997; Wood & Simons, 2018) and that a large illusion-inducing rectangle, although never consciously noticed, still affected the reported position of a target (Lathrop, Bridgeman, & Tseng, 2011). Jiang and Leung (2005) provided evidence that latent learning of repeated information occurred without attention and facilitated performance in a later performed task. Also, imaging methods were able to demonstrate substantial neuronal marks of stimuli that remain beneath the threshold of awareness due to inattentional blindness (Pitts, Martínez, & Hillyard, 2012): Amplitudes of early event-related potentials (ERPs) that were recorded over the occipital pole differentiated between visually presented shapes and random arrays regardless of whether or not participants were aware of the shapes. Finally, there are even hints in inattentional blindness research indicating that the processing of objects that remain undetected goes beyond mere perceptual processing: A recent study provides direct evidence for the conclusion that the semantics of the undetected stimuli can be processed preconsciously; in two experiments, reaction times for target stimuli in a primary task were significantly slower when the semantic content of an undetected stimulus contradicted that of the attended, to-be-judged stimulus (Schnuerch, Kreitz, Gibbons, & Memmert. 2016; for indirect evidence see also Calvillo & Jackson, 2014; Koivisto & Revonsuo, 2009).

Summed up, to this point evidence suggests that unattended and unnoticed stimuli during inattentional blindness can be processed to some degree. It remains unclear however, which factors determine the occurrence and strength of such preconscious processing. One influencing factor that has already been shown to determine whether or not inattentional blindness occurs, and that could, therefore, play a role in preconscious processing of unnoticed objects as well, is the perceptual load of the primary task. The perceptual load model (e.g., Lavie, 1995) suggests that the processing of task-irrelevant stimuli depends on the perceptual load of the primary task; low perceptual load of the attended information leaves free attentional capacities that can spill over to task-irrelevant stimuli. In contrast, high load captured more attentional resources and prevents the processing of irrelevant stimuli. This model was tested and supported by a variety of empirical data, mainly demonstrating an influence on reaction times (e.g., Furley, Memmert, & Schmid, 2013; Lavie, 1995, 2005; Lavie & Fox, 2000; Lavie, Hirst, de Fockert, & Viding, 2004). Some studies already investigated the influence of perceptual load on whether or not an unexpected object crosses the threshold of awareness (i.e., was reported by the participants). And indeed, this line of research suggests that unexpected and unattended stimuli are more likely to be detected when the perceptual load of the primary task is low (Calvillo & Jackson, 2014; Cartwright-Finch & Lavie, 2007; but see Koivisto & Revonsuo, 2009). In a more applied setting, high perceptual load also significantly increased inattentional blindness and reaction times to hazards in a driving simulator (Murphy & Greene, 2016, 2017). The question that remains is whether perceptual load also moderates the processing of stimuli that do not receive voluntary attention and thus go unnoticed in an inattentional blindness setting.

As argued above, previous theorizing and empirical data suggest that the processing of unexpected objects is a continuous process that oversteps the binary threshold of awareness at some point, but can also be measured beneath that threshold. We therefore hypothesize that those factors that influence whether or not a stimulus crosses the threshold of awareness also determine the degree of preconscious processing, and that one of these factors might be the perceptual load of the primary task. To investigate this question, we adapted a recently established inattentional blindness paradigm that uncovered preconscious processing of unattended and undetected stimuli through an interference effect measured by reaction times (Schnuerch et al., 2016). We systematically varied perceptual load of the primary task to test the assumption that predictions of the perceptual load model also hold true for stimuli that remain unconscious due to inattentional blindness. The present study will not only expand knowledge on inattentional blindness but will also provide evidence for the generalizability of the perceptual load model to the processing of stimuli that remain unconscious.

## Experiment 1

The first experiment examined whether higher perceptual load of a primary task reduces preconscious processing of unexpected stimuli to which participants are inattentionally blind. The employed method was based on the inattentional blindness paradigm developed by Schnuerch et al. (2016), in which the unexpected and unattended stimuli are presented repeatedly, and reaction times are used as dependent variables. More precisely, participants had to respond as fast and as accurately as possible to target numbers while simultaneously being presented with unexpected numbers. Those unexpected and unattended numbers were either congruent or incongruent with the target number. Participants’ target response was significantly slower when incongruent stimuli were present simultaneously. In our understanding, the original paradigm of Schnuerch et al. (2016) incorporates only low perceptual load in the primary task as there were no distractors involved in the primary task. In the present study, we expanded the paradigm by a high-load condition. In accordance with the perceptual load model, we expected to replicate the interference effect found by Schnuerch et al. (2016) in the low-load condition, as this condition should leave enough attentional resources to additionally process the unexpected and unattended numbers. In contrast, we expected the interference effect to diminish in trials of higher perceptual load.

### Method

Experiment 1 was registered on the Open Science Framework prior to data collection. Hypotheses, procedure, sample size, exclusion criteria, data preparation, and analyses were specified in advance and are available online along with the data (https://osf.io/zevup/). All procedures were in accordance with the revised 2013 Helsinki Declaration and have been approved by the local ethics committee.

#### Participants

We used the software G*Power 3.1.6 (Faul, Erdfelder, Lang, & Buchner, 2007) to calculate a priori power for our experiment. Presuming a small-to-medium effect size of *f*(*U*) = 0.3, coupled with a critical alpha of .05 and seeking for a high power level of 1 − β = 0.95, the analysis indicated a total sample size of 57 participants to be needed for a repeated-measures ANOVA, with one group and four within factors. Expecting an exclusion rate of up to 30% (see Schnuerch et al., 2016), we collected a total sample of 80 participants for Experiment 1, to ascertain a sufficient level of power.

The sample was recruited from courses, on campus, and via online networks of the German Sports University Cologne. All participants gave written informed consent, received monetary compensation, and were debriefed after the experiment. Data from 65 participants were analyzed (*M*_age_ = 21.5 years, *SD*_age_ = 2.4 years, 47.7% female). From the 80 participants that were initially tested, we excluded 15 participants from the analysis as they (a) did not have normal or corrected-to-normal vision (visual acuity trials; zero participants), (b) anticipated the unexpected object or knew that inattentional blindness was the subject of the study (zero participant), (c) noticed the unexpected and unattended numbers during the inattentional blindness phase (11 participants), and/or (d) did not notice the before unexpected and unattended stimulus in the control condition in which they did not have to perform the primary task any more in at least 80% of the trials (full-attention trials; four participants). One participant was excluded because of a technical error during the data collection.^2^

#### Materials and procedure

During the whole experiment, participants were seated in front of a 24-inch display monitor with a resolution of 1,920 × 1,080 pixels and a standardized distance of 60 cm from the screen, which was secured by using a chin rest. The inattentional-blindness task was programmed and run on Presentation (Neurobehavioral Systems, Albany, NY). Participants were tested alone or in pairs (work spaces were separated by dividers). After the inattentional-blindness task, participants filled out a questionnaire collecting demographics, anticipation of an unexpected object, and general knowledge about inattentional blindness. Afterward, they were debriefed and compensated.

**Fig. 1:**
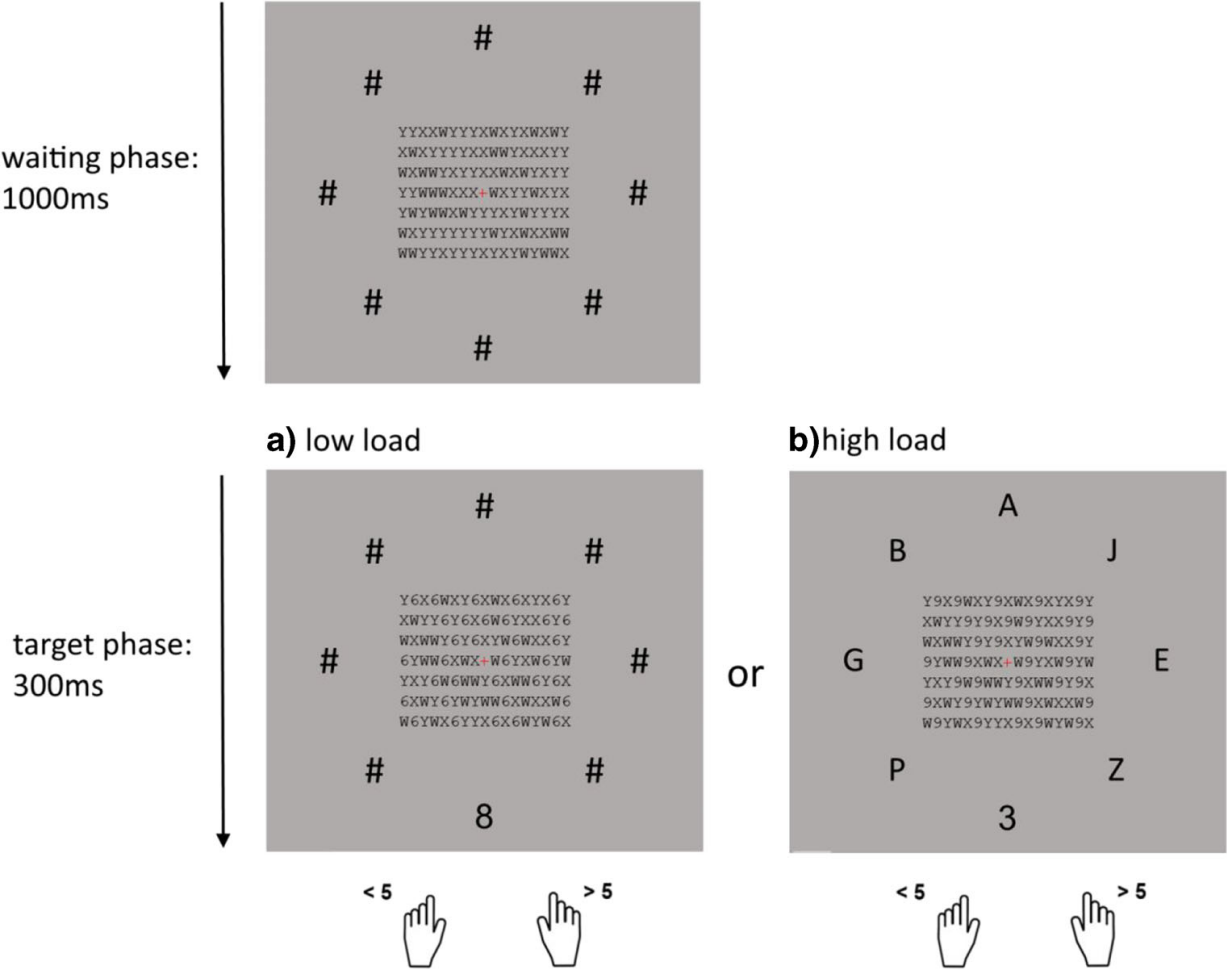
Schematic of the experimental task in Experiment 1. Participants categorized the peripheral number as smaller or greater than 5 as quickly as possible. Every trial consisted of two phases: waiting phase and target phase. In the target phase, the target number appeared at one of the eight positions of the hashtags. In half of the trials (**a:** low-load condition) the other hashtags stayed the same while in the other half of the trials (**b:** high-load conditions) the other hashtags change to letters. In the irrelevant central array half of the characters were numbers matching the category of the peripheral, to-be-judged numeral (**a:** congruent condition) or pertaining to the opposite category (**b:** incongruent condition)

Our design was, on the one hand, based on a paradigm previously introduced by Schnuerch et al. (2016) to examine preconscious processing under inattentional blindness, and, on the other hand, based on paradigms used to investigate perceptual load (Lavie, Beck, & Konstantinou, 2014). Each experimental trial consisted of two phases: the waiting phase and the target phase. For the entire trial, participants continuously fixated on a small red cross in the centre of the screen. Around the fixation cross black letters (*X, Y, W*) were located in seven rows with 17 letters each. This letter square was not relevant for the primary task. In each phase of a trial the type of letter (*X, Y,* or *W*) for each position was randomly drawn (within the target phase of the experimental trials half of the letters were unexpectedly replaced by numbers instead of an *X, Y,* or *W*). Within the waiting phase, eight black hashtag symbols (#) were located equally spaced on an imaginary circle with a radius of 200 pixels around the central square of letters. This waiting screen was shown for 1,000 ms. Afterward, at the location of one of the eight #-symbol, the target number appears (1, 2, 3, 4 or 6, 7, 8, 9) for 300 ms (see Fig. 1). Participants were instructed to press a predefined key if the number is smaller than five and another key if the number is greater than five. They were asked to react as quickly and as accurately as possible. In 80 out of 160 trials, the target number appeared at the location of one of the #-symbols. This constituted the low-load condition (see Fig. 1a). In the remaining 80 trials, the target number appeared as well at one out of the eight #-symbol positions, while the other positions were filled with a random letter (see Fig. 1b). This constituted the high-load condition. Simultaneously, the unattended letters (*X, Y,* and *W*) in the letter array around the fixation cross changed: In all 160 trials, unexpectedly, half of these letters were not randomly replaced by another letter, but by a number (1, 2, 3, 4 or 6, 7, 8, 9). In 50% of these trials, this unexpected and unattended number matched the category of the target number (congruent trial). In the other 50% of the trials, the number mismatched the category of the target number (incongruent trial). Participants were asked to respond as quickly and as accurately as possible and decide whether the target number was smaller or greater than five. They had a maximum time interval of 2,000 ms to respond. After 300-ms presentation of the target screen, a blank screen was shown. If a response was made, the next trial started immediately.

At the beginning of the experiment, participants performed 20 practice trials and received feedback about the number of correct responses and their average reaction time. This practice block was repeated until at least 17 correct responses were made. Afterward, participants had to complete 160 experimental trials (which was chosen based on Schnuerch et al., 2016, used number of trials to achieve sufficient reliability). Trial types were randomly intermixed, but added up to 40 trials per condition (congruent/incongruent combined with low/high load). After every 27 trials, participants received feedback about their performance (% correct responses, mean reaction time) and were able to take a break for as long as they wanted. After completion of the experimental trials, participants were asked whether they had noticed anything around the central fixation cross other than the three letters *X, Y,* and *W*. If they responded yes, they were further asked to describe in detail what they noticed and how many times. Participants’ who reported seeing numerals at least once during the experiment were excluded from all further analyses because the processing of the numbers could then no longer unambiguously be defined as preconscious.

Afterward, participants completed a block of 20 control trials (full-attention trials). These control trials were identical to the critical experimental trials, except that participants were instructed to lo longer attend to the circle of hashes and the target number. After each trial, they were asked to report the number presented in the central square around the fixation cross (not the target number in the circle of hashes, but the number in the central letter square). At the end, participants were tested for their visual acuity to ensure normal or corrected-to-normal sight. For that, they completed 10 control trials in which they had to identify numbers on the display with the same font and size as the unattended numbers in the trials before.

### Results and discussion

A 2 × 2 repeated-measures analysis of variance (ANOVA) of mean reaction times with the two-level factors “condition” (congruent, incongruent) and “load” (low load, high load) was conducted. Partial eta-squared (${\eta}_P^2$) is reported as a measure of effect size for the ANOVA.^3^

**Fig. 2:**
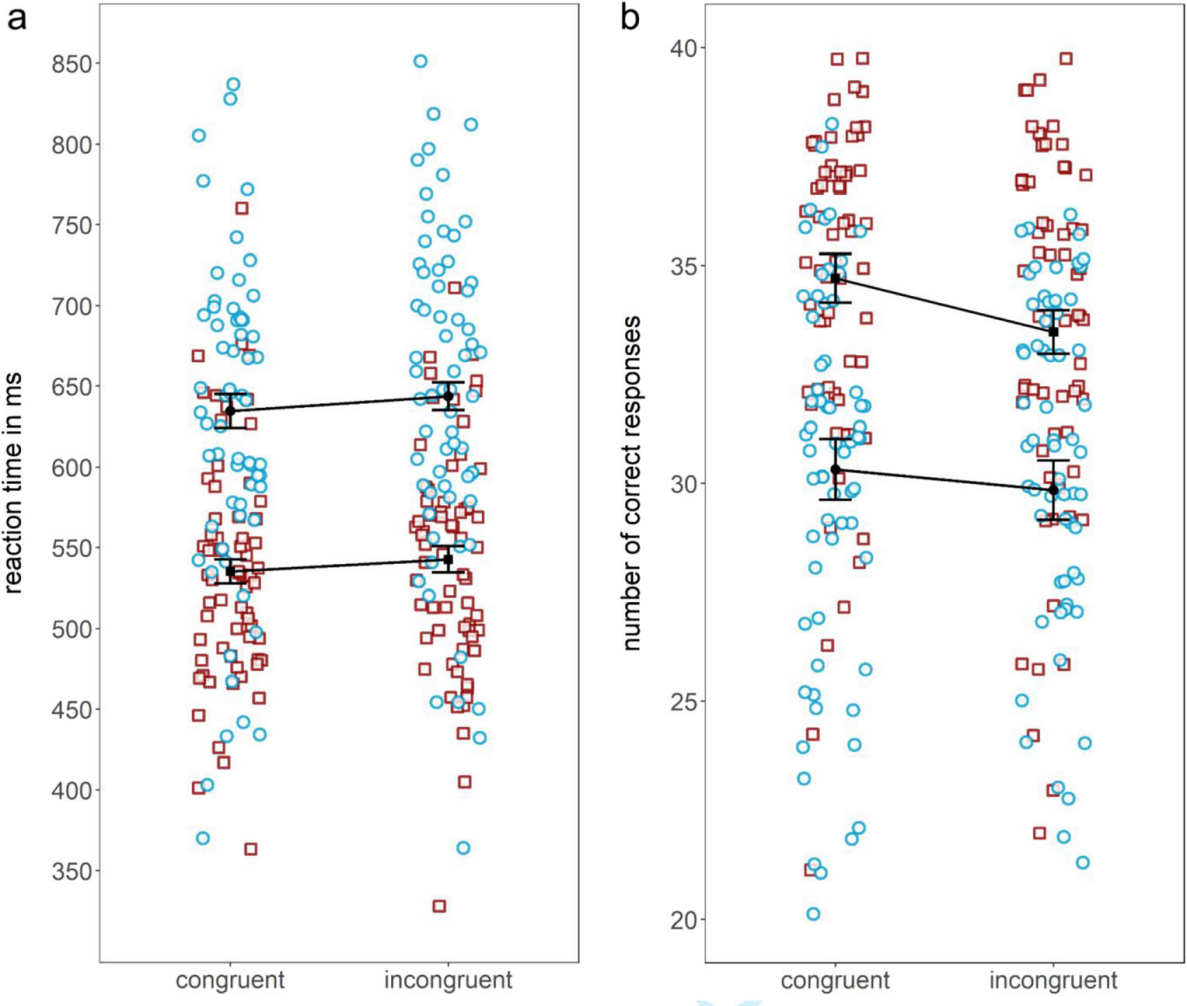
Means (**a:** reaction times and **b:** number of correct reactions [max = 40]) and individual data points for each of the four experimental conditions in Experiment 1 (□ = low-load condition; ○ = high-load condition). Error bars depict the 95% confidence interval

The conducted ANOVA revealed a significant main effect of condition, with significantly higher mean reaction times in the incongruent condition than in the congruent condition, *F*(1, 64) = 7.40, *p* < .01, ${\eta}_P^2$ = .10. This replicates the interference effect found by Schnuerch et al. (2016) and generalized it to another variant of the paradigm. Furthermore, a significant main effect of perceptual load was found, with significantly higher mean reaction times in the high-load condition than in the low- load condition, *F*(1, 64) = 266.69, *p* < .001, ${\eta}_P^2$ = .81. This effect has been interpreted as successful manipulation of load in previous load research (Lavie et al., 2004). However, against our expectations, no significant interaction effect of load and condition was found, *F*(1, 64) = 0.07, *p* = .80, ${\eta}_P^2$ = .001^4^ (see Fig. 2). To investigate whether this pattern of results also occurs with error rates, we performed an exploratory analysis with number of correct reactions in the primary task as dependent variable. The pattern of results was identical: We found a significant main effect of condition, with significantly higher mean reaction times in the incongruent condition than in the congruent condition, *F*(1, 64) = 7.84, *p* < .01, ${\eta}_P^2$ = .11, as well as of load, with significantly higher mean reaction times in the high-load condition than in the low-load condition, *F*(1, 64) = 169.48, *p* < .001, ${\eta}_P^2$ = .73, but no interaction effect, *F*(1, 64) = 1.47, *p* = .23, ${\eta}_P^2$ = .02 (see Fig. 2). Means and standard deviations for reaction times and the number of correct reactions for Experiment 1 are reported in Table 1. The results indicate that preconscious processing of the unattended numbers did not only influence response times but also the accuracy of responses.

**Table 1:** Summary statistics for Experiment 1

	*M* (*SD*) of mean reaction time		*M* (*SD*) of number of correct reactions (out of 40)	
	Congruent	Incongruent	Congruent	Incongruent
Low load	535.61 (72.01)	543.38 (69.38)	34.71 (4.01)	33.48 (4.27)
High load	635.12 (113.38)	644.52 (100.39)	30.32 (4.53)	29.85 (4.74)

*Note*. *M* = mean; *SD* = standard deviation

Replicating the hypothesized main effects of condition and load demonstrates the adequacy and sensitivity of the implemented design. Nevertheless, we did not find the hypothesized interaction effect; the interference effect (effect of condition) does not seems to be modulated by perceptual load. We are aware, however, that null hypothesis significance testing cannot provide evidence for the absence of an effect. Bayes factors, on the other hand, can quantify the relative support for a null model over other models (Harms & Lakens, 2018). Thus, we additionally performed a Bayesian repeated-measure ANOVA (*r* scale for fixed effects = .5 and for random effects = 1) with JASP (JASP Team, 2017) to further examine the nonsignificant interaction effect. Bayes factors indicated that it is 3 to 6 times more likely that there is no effect than that there is the specified interaction effect for both, mean reaction time (BF01 = 5.49) and number of correct reactions (BF01 = 2.94). Nevertheless, Bayes factors of this size constitute only an indication rather than strong evidence for the null hypothesis (Kass & Raftery, 1995). Thus, further investigation seemed warranted to replicate the finding that perceptual load does not modulate preconscious processing.

## Experiment 2

To strengthen the findings from Experiment 1 and to generalize them to different task settings, we carried out a conceptual replication with a slightly different experimental design. As the effect sizes for the interference effect (effect of condition) in Experiment 1 were smaller than in the original study by Schnuerch et al. (2016), we decided to (a) test even more participants in Experiment 2 and (b) to adapt the design closer to the original paradigm by Schnuerch et al. (2016). Thus, in Experiment 2, we induced perceptual load by increasing the number of distractors in direct spatial proximity of the target instead of spreading them across the whole display.

### Method

Experiment 2 was also registered on the Open Science Framework prior to data collection. Hypotheses, procedure, sample size, exclusion criteria, data preparation, and analyses were specified in advance and are available online along with the data (https://osf.io/vtbdq/).

#### Participants

Taking the null results of Experiment 1 into account, we decided to adapt the a priori power analysis for Experiment 2 to achieve high power even with smaller effects than expected before. In detail, presuming a small effect size of *f*(*U*) = 0.25 now, coupled with a critical alpha of .05 and seeking for a high power level of (1 − β) = 0.95, the analysis indicated a total sample size of 94 participants needed for a repeated-measures ANOVA, with one group and four within factors. As we learned that the exclusion rate in inattentional blindness studies cannot be reliably predicted, we decided to test participants until at least 94 would fit the inclusion criteria.

Again, the sample was recruited from courses, on campus, and via online networks of the German Sports University Cologne. All participants gave written informed consent, received monetary compensation, and were debriefed after the experiment. Data from 102 participants were analysed (*M*_age_ = 20.4 years, *SD*_age_ = 2.1 years, 52.9% female). From the 114 participants that were initially tested, we excluded 12 participants from the analysis on the same exclusion criteria as in Experiment 1, when they (a) did not have normal or corrected-to-normal vision (visual acuity trials; 0 participants), (b) anticipated the unexpected object or knew that inattentional blindness was the subject of the study (two participants), (c) noticed the unexpected and unattended numbers during the inattentional blindness phase (4four participants), or (d) did not notice the before unexpected and unattended stimulus in the control condition in which they no longer had to perform the primary task in at least 80% of the trials (full-attention trials; four participants). Two participants were excluded because of a technical error during the data collection.

#### Materials and procedure

**Fig. 3:**
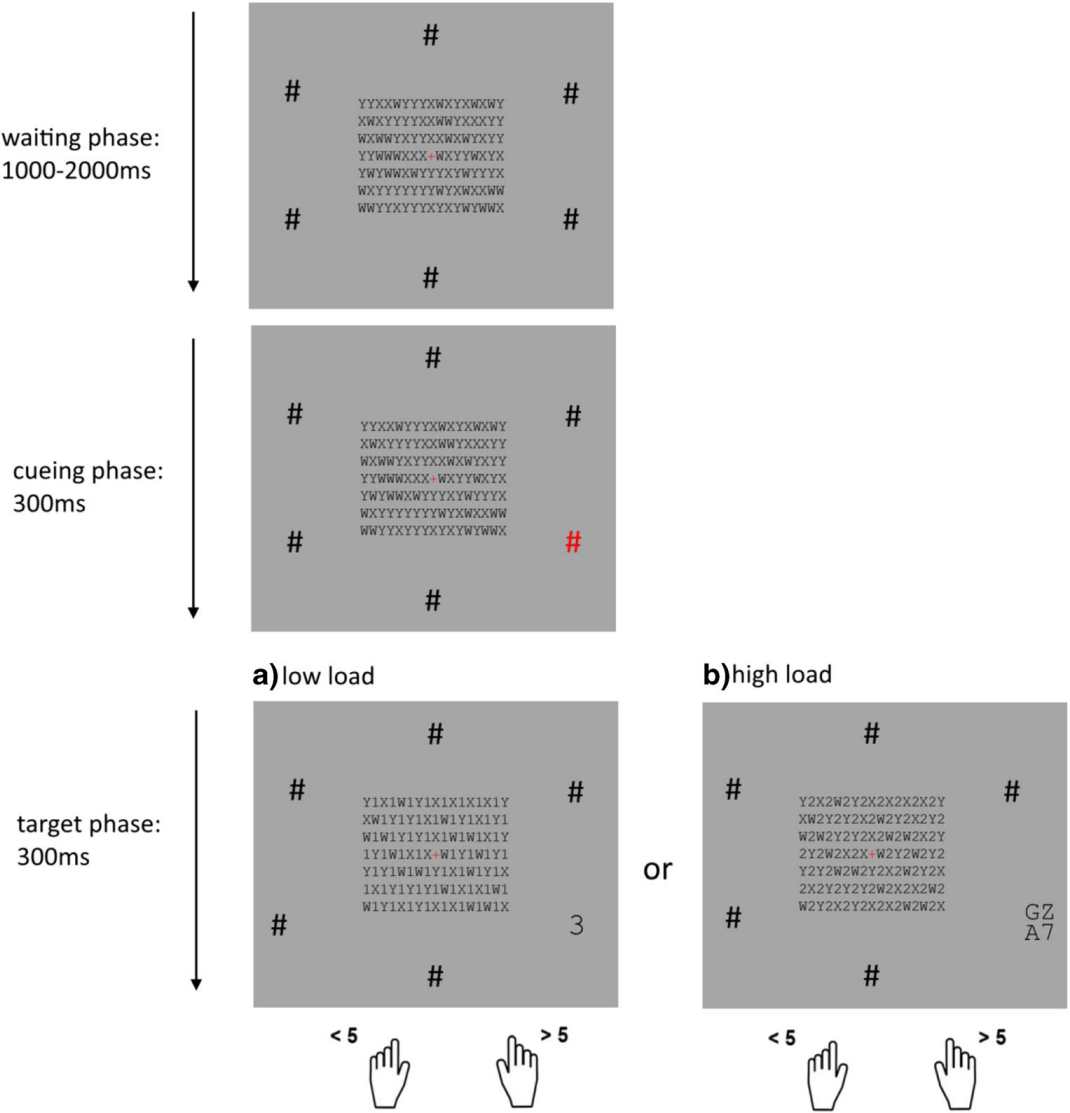
Schematic of the experimental task in Experiment 2. Participants categorized the peripheral number as smaller or greater than 5 as quickly as possible. Every trial consisted of three phases: waiting phase, cueing phase, and target phase. In the target phase, in half of the trials the target number appeared alone (**a:** low-load condition) and in half of the trials it appeared with three distractor letters (**b:** high-load conditions). In the irrelevant central array half of the characters were numbers matching the category of the peripheral, to-be-judged numeral (**a:** congruent condition) or pertaining to the opposite category (**b:** incongruent condition)

The experimental protocol and procedure of Experiment 2 was identical to that of Experiment 1, except for small differences in the design of the inattentional blindness task: Each trial now consisted of three phases: the waiting phase, the cueing phase, and the target phase. The letter arrays in the centre of the screen around the fixation stayed identical to Experiment 1. Around the central square of letters, six instead of eight black hashtag symbols (#) were located, equally spaced on an imaginary circle, to have more space at each location for the additional distractor letters (see Fig. 3). In the waiting phase, the described screen was presented for a random interval between 1,000 and 2,000 ms. In the cueing phase, one of the six hashtags changed from black to red for 300 ms, which constituted a 100% valid cue for the location of the following target. Afterward, in the target phase, the target number appeared (1, 2, 3, 4 or 6, 7, 8, 9) for 400 ms at the location of the red hash. In the low-load condition (50% of the experimental trials), the target number appeared alone at one out of four positions at the location of the red hash (see Fig. 3a). In the high-load condition, the target number appeared at one out of four positions, while the other three positions were randomly filled with letters (see Fig. 3b). Participants had the same instructions as in Experiment 1—namely, to categorize the number as smaller or greater than 5 as quickly and accurately as possible.

### Results and discussion

**Fig. 4:**
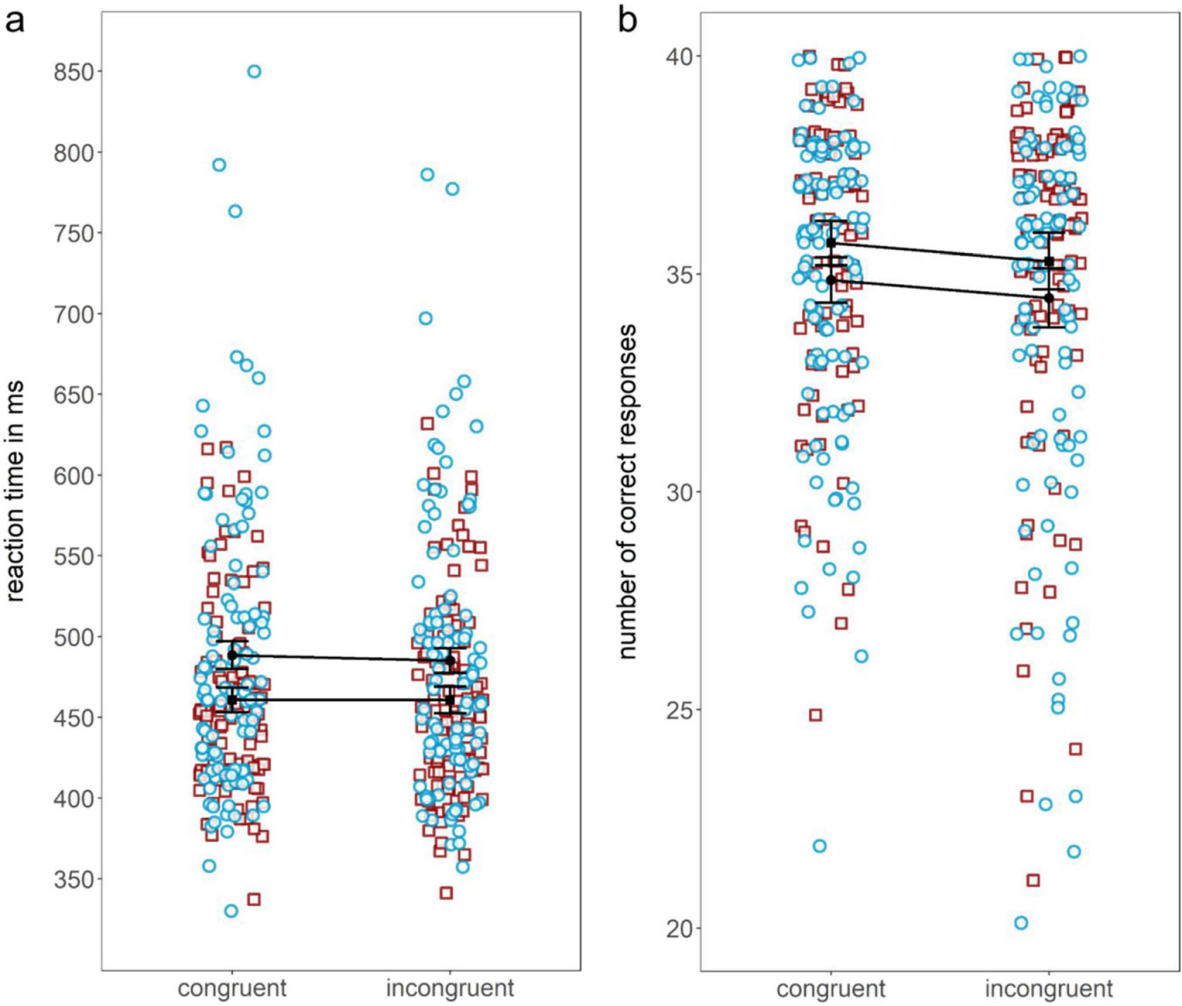
Means (a: reaction times and b: number of correct reactions [max = 40]) and individual data points for each of the four experimental conditions in Experiment 2 (□ = low-load condition; ○ = high-load condition). Error bars depict the 95% confidence interval

As in Experiment 1, we ran a 2 × 2 repeated-measures ANOVA of mean reaction times with the two-level factors “condition” (congruent, incongruent) and “load” (low load, high load).^5^ Replicating the findings from Experiment 1, there was no significant interaction effect, *F*(1, 101) = 0.92, *p* =.34, ${\eta}_P^2$ = .01. There was, however, a significant main effect of load, with significantly longer reaction times in the high-load compared with low-load trials, *F*(1, 101) = 15.51, *p* < .001, ${\eta}_P^2$ = .13. Unexpectedly and in contrast to Experiment 1, the ANOVA revealed no significant main effect of condition, *F*(1, 101) = 1.12, *p* = .29, ${\eta}_P^2$ = .01.^6^ Although the experimental design has been even closer to the original paradigm of Schnuerch et al. (2016) than the experimental design of Experiment 1 and despite an even higher power, we could not replicate an interference effect for reaction times. However, an analysis of response accuracy instead of response times replicates the exact same pattern from Experiment 1: A repeated-measures ANOVA, with the number of correct reactions in the primary task as dependent variable, showed a significant main effect of load, *F*(1, 101) = 3.94, *p* < .05, ${\eta}_P^2$ = .04, as well as condition, *F*(1, 101) = 4.26, *p* < .05, ${\eta}_P^2$ = .04. The number of correct reactions was significantly higher in the low-load condition than in the high-load condition and significantly higher in the congruent than in the incongruent condition. Again, no interaction effect was found, *F*(1, 101) < 0.01, *p* = .99, ${\eta}_P^2$ < .001 (see Fig. 4). Means and standard deviations for reaction times and the number of correct reactions for Experiment 2 are reported in Table 2.

**Table 2:** Summary statistics for Experiment 2

	*M* (*SD*) of mean reaction time		*M* (*SD*) of number of correct reactions (out of 40)	
	Congruent	Incongruent	Congruent	Incongruent
Low Load	461.38 (57.89)	461.29 (60.56)	35.71 (3.10)	35.29 (3.82)
High Load	489.23 (92.27)	485.89 (84.68)	34.86 (3.48)	34.45 (4.55)

*Note*. *M* = mean; *SD* = standard deviation

To further examine the nonsignificant interaction effects, we performed a Bayesian repeated-measure ANOVA (*r* scale for fixed effects = .5 and for random effects = 1). Bayes factors showed substantial evidence in favour of the H0 over the H1 (Kass & Raftery, 1995), as they indicated that it is 6 times more likely that there is no effect (null model) than there is the specified interaction effect for both, number of correct reactions (BF01 = 6.33) and mean reaction times (BF01 = 6.24). Results from the individual Bayesian analyses are further supported by estimating Bayes factors for the combined data of both experiments (BF01 = 7.14 for the number of correct reactions and BF01 = 7.58 for mean reaction times). Thus, against our hypothesis, but in accordance with our findings from Experiment 1, the interference effect does not seem to be modulated by perceptual load; our analyses did not reveal an interaction between these two factors.

## General discussion

In two experiments, we investigated whether perceptual load of a primary task affects the processing of unattended stimuli that remain beneath the threshold of awareness. Perceptual load has repeatedly been demonstrated to affect task-irrelevant stimulus processing (for a review, see Lavie et al., 2014). Previous work that varied perceptual load in an inattentional blindness setting has so far focussed on the question of how perceptual load influences whether or not people notice unexpected and unattended objects (Calvillo & Jackson, 2014; Cartwright-Finch & Lavie, 2007; Koivisto & Revonsuo, 2009). As most of those studies demonstrated that high perceptual load of the attended information increases susceptibility to inattentional blindness, we expected high perceptual load in a primary task to also reduce preconscious processing of unattended stimuli, indicated by a diminishing interference effect. However, against our expectations, high perceptual load in the primary task had no significant impact on preconscious processing of unattended stimuli, as it did not reduce the interference effect.

Our results might be interpreted in two ways: (a) Our null findings might indicate that our study design was not suitable to test the raised hypothesis, or (b) our design was suitable, but the hypothesized effect might not exist. We will first discuss interpretation *a*.

Several considerations speak against that interpretation. First, we used reaction times and the number of correct reactions to operationalize processing instead of a report/no-report paradigm. Also, we based our analysis on a total of 160 trials instead of only focusing on one critical trial, as is done in traditional inattentional blindness studies. Thus, our design should have been sensitive to changes in processing and definitely a more sensitive measure of stimulus processing than classical inattentional blindness paradigms.

Second, for both experiments, a priori power analyses ensured sufficient power and indicated that even small effects should have been detected. Additionally, we were able to replicate our findings in a second experiment with an even higher power. Thus, our null effects cannot be easily explained by a lack of statistical power. Further, Bayes factors allowed us a more informative investigation of the null effects by quantifying the relative evidence in the data for a null model compared with the alternative model. Even though there are no statistical techniques that can unconditionally prove the null hypothesis the combined evidence of our experiments was in favor of a nonexisting interaction effect.

Third, we were able to establish necessary prerequisites to also potentially find an interaction effect: In both experiments, we found a significant effect of load on the primary task performance. Thus, we demonstrated that our manipulation of load worked. The absence of a modulating effect of load on the interference effect does not hinge on inadequate implementation of perceptual load in our design.

Also, in both experiments we found a significant interference effect. When searching for a reduction of the interference effect by higher load, the effect itself must of course be present in the first place. In Experiment 1, reaction times were slower when the unnoticed stimuli were incongruent to the target stimuli than when they were congruent. Also, the number of correct reactions was reduced in incongruent trials. Thus, the interference effect found by Schnuerch et al. (2016) was replicated and extended with a different experimental design in the present study. Unfortunately, in Experiment 2, the interference effect was only significant for the number of correct reactions, but not for the mean reaction times. This was especially surprising as (a) response times are in general more sensitive than response accuracy, and (b) the experimental design of Experiment 2 was more similar to the original study of Schnuerch et al. (2016), in which the interference effect was found in two experiments. This prerequisite means that the missing interaction effect for reaction times cannot be interpreted in Experiment 2. We did, however, find a significant interference effect for accuracy, and this effect was also not modulated by load.

Summed up, our findings suggest that perceptual load might not affect the processing of unexpected and unnoticed stimuli that remain beneath the threshold of awareness (explanation *b*). Even under high load (main effects of load were of large effect size in both studies), task-irrelevant stimuli are still processed to an extent that enables them to affect performance in a primary task. This supports the idea of automatic processing, which covers the whole visual field (and not only the centre of attention) and which is not significantly affected by the overall perceptual load of the task-relevant information. Bressan and Pizzighello (2008) also proposed that an object that goes unnoticed can still cause a state of alert, which in turn can generate an attentional shift. This attentional shift to the unexpected and unattended object can get prevented by an attention consuming task, though. However, the attention-consuming task does not prohibit that a portion of the attentional resources is allocated to the object, which can be strong enough to cause a disturbance in primary task performance (Bressan & Pizzighello, 2008).

This interpretation of the data raises the question as to why some studies were able to find effects of perceptual load on whether or not an unattended object crosses the threshold of awareness (Calvillo & Jackson, 2014; Murphy & Greene, 2016). Is it realistic to assume that a variable influences the passing of a threshold, but not the amount of processing beneath that threshold? Taking a closer look at those studies that found effects of perceptual load on the processing of unexpected and unattended objects (Calvillo & Jackson, 2014; Murphy & Greene, 2016) and those that did not (Koivisto & Revonsuo, 2009; Lathrop et al., 2011) indicates that the answer might be found in the relation between the attended information and the unexpected and unattended stimuli. Koivisto and Revonsuo (2009) demonstrated that task-irrelevant stimuli enter awareness irrespective of perceptual load when their meaning is relevant to the observer’s task (i.e., attentional set; Folk, Remington, & Johnston, 1992). Due to their relevance, these objects are processed sufficiently to be detected despite an attention-consuming task. Perhaps the high similarity of unexpected and unattended stimuli and target stimuli in the present study (both were numerals) has increased the relevance of the unexpected stimuli. Hence, the unexpected stimuli got processed enough to cause an interference effect even under a high amount of perceptual load in the primary task.

While focusing on a task, an attentional set for task-relevant information is formed (Folk et al., 1992). Based on the previously described findings and theorizing, we assume that an automatic processing of the whole visual field might uncover unexpected stimuli which, if they share features with the current attentional set, are allocated more attentional resources. All available attentional resources can be focused on the task-relevant information and, as predicted by the perceptual load model, only spill over to unattended stimuli under conditions of low perceptual load, but less or not at all under high perceptual load. Thus, the attentional spill-over under low perceptual load can allow a deeper processing of task-irrelevant stimuli. The combination of earlier processing of the task-irrelevant stimuli, due to their similarity to the current attentional set, may lead to an attentional shift that causes the stimulus to exceed the threshold of awareness (e.g., Calvillo & Jackson, 2014; Murphy & Greene, 2016).

Finally, we want to raise awareness about whether the failure of participants to report the unexpected stimulus is, as assumed, due to a perceptual failure or due to a failure of memory. The latter interpretation suggests that inattentional blindness can sometimes be inattentional amnesia, proposing that people do indeed consciously process the unexpected stimulus, but then forget about it until being queried about it (Wolfe, 1999). As the critical object has to be completely unexpected by definition, participants cannot be queried multiple times about it. This makes a direct investigation of this question very difficult. However, indirect evidence makes the explanation of inattentional amnesia rather unlikely: There were no improvements in detection rates when stopping the experiment and asking about the unexpected stimulus immediately after it appeared rather than later (Becklen & Cervone, 1983) or by using astonishing unexpected objects that seem hard to forget once detected (Simons & Chabris, 1999). We used an operationalization that has, in similar forms, been successfully implemented in other research endeavours (e.g., Pitts et al., 2012; Schnuerch et al., 2016) and seems the best approximation for our research question. However, while we can conclude that preconscious processing takes place and that this preconscious processing can influence overt behaviour (reaction times and accuracy), and that this processing is not modulated by perceptual load, we want to point out that we cannot define this processing at this time. It seems to be a process that hinders verbal report and can adopt different levels beyond that reportability, but further research would be needed to investigate the nature of this processing in depth.

### Conclusion

Earlier studies investigating the perceptual load model using the classical binary inattentional blindness paradigm provide mixed results for the question of whether awareness of a task-irrelevant stimulus gets significantly reduced by higher perceptual load (Calvillo & Jackson, 2014; Cartwright-Finch & Lavie, 2007), or not (Koivisto & Revonsuo, 2009; Lathrop et al., 2011). Our study adds a new piece of knowledge to previous research by looking at preconscious processes. Our results suggest that task-irrelevant stimuli might be allocated to more attentional resources than originally assumed by the perceptual load model if they belong to the attentional set of the primary task. Therefore, preconscious processing of such unexpected stimuli might not be primarily depend on perceptual load, but dominantly on their relevance for the task at hand (Koivisto & Revonsuo, 2009; Kreitz et al., 2016; Memmert, 2006; Most et al., 2001). These findings are in line with results demonstrating that even expectations are subordinate to an attentional set in the context of inattentional blindness (Kreitz et al., 2016). More research is needed to explore the mechanisms of stimuli processing beneath the threshold of awareness in general and in relation to the perceptual load model.

## Footnotes

1 We use this taxonomy of Dehaene et al. (2006) to denominate the type of processing discussed in the present manuscript. By using the term *preconscious,* we want to specify the overarching term *unconscious* and, thereby, clearly distinguish processing of critical stimuli despite inattentional blindness (i.e., stimuli with sufficient bottom-up strength but no top-down amplification) from subliminal processing (i.e., attended stimuli with weak bottom-up strength).

2 Numbers do not add up neatly, as some participants met more than one exclusion criterium.

3 As we used a minimum reaction time of 100 ms as cut off criteria to exclude accidental reactions, three trials (all from one participant) were excluded a priori.

4 Using median reaction times as dependent variable for the 2 × 2 repeated-measures analysis of variance provided a similar pattern of results, showing a significant main effect of load, *F*(1, 64) = 248.57, *p* < .001, ${\eta}_P^2$ = .80, and a significant main effect of condition, *F*(1, 64) = 5.86, *p* < .05, ${\eta}_P^2$ = .08, but no interaction effect, *F*(1, 64) = 0.01, *p* =.91, ${\eta}_P^2$ < .001.

5 As we used a minimum reaction time of 100 ms as cutoff criteria to exclude accidental reactions, in total 15 trials (from eight participants) were excluded a priori.

6 Using median reaction times as dependent variable for the 2 × 2 repeated-measures analysis of variance provided a similar pattern of results, showing a significant main effect of load, *F*(1, 101) = 16.72, *p* < .001, ${\eta}_P^2$ = .14, but no main effect of condition, *F*(1, 101) = 0.88, *p* =.35, ${\eta}_P^2$ = .01, and no interaction effect, *F*(1, 101) = 1.13, *p* =.29, ${\eta}_P^2$ = .01.
